# The Effect of Hydrated Lime on the Low-Temperature Properties of Foamed Asphalt Mixture (FAM)

**DOI:** 10.3390/ma19153219

**Published:** 2026-07-28

**Authors:** Mateusz Marek Iwański, Małgorzata Cholewińska, Marcin Podsiadło

**Affiliations:** 1 Department of Construction Engineering, Kielce University of Technology, 25-341 Kielce, Poland; 2 Department of Transportation Engineering, Kielce University of Technology, 25-341 Kielce, Poland; m.cholewinska@tu.kielce.pl; 3 Faculty of Civil Engineering and Architecture, Kielce University of Technology, 25-341 Kielce, Poland; mpodsiadlo@tu.kielce.pl

**Keywords:** foamed asphalt mixture, surface-active agent, hydrated lime, foamed asphalt binder, crack propagation, resistance to low-temperature cracking, resistance to permanent deformation

## Abstract

**Highlights:**

- Foamed asphalt binder with SAA and lime provide resistance to moisture, frost and high temperatures of the FAM.
- The synergy of foamed asphalt binder with SAA and lime provides resistance to low-temperature cracking of the FAM.
- The optimal content of foamed asphalt binder with SAA and hydrated lime in the FAM was determined.

**Abstract:**

Foamed asphalt mixtures (FAMs) are considered to be among the most environmentally friendly. They are produced at temperatures ranging from 100 °C to 120 °C. In order to produce asphalt mixtures at such a low temperature, it is necessary to produce foamed asphalt binder with high foaming parameters, i.e., maximum expansion (ER) and a half-life (HLa) of the asphalt foam. Consequently, the asphalt binders were modified with a surfactant at a concentration of 0.6% by weight of the binder, prior to its foaming with water. Subsequently, an AC 8 S asphalt mixture was designed using traditional hot-mix asphalt (HMA) technology and with modified foamed asphalt binders in quantities ranging from 5.6% to 6.5% by weight, in increments of 0.3%. To ensure optimal properties of the FAM, hydrated lime was added at levels of 0%, 15%, 30% and 45% by weight as a substitute for filler. The influence of modified foamed asphalt binders and hydrated lime on the void content (Va), resistance to moisture and frost (TSR) and resistance to permanent deformation (WTS_AIR_ and PRD_AIR_) of the FAM was assessed. A key element of the research was the determination of the complex modulus of stiffness E* and resistance to low-temperature cracking R_−2_, σ_cry_, T_failure_ and crack propagation using the SCB methodology. Analysis of the test results using desirability functions enabled the determination of the optimum proportions of foamed asphalt binders and hydrated lime—5.9% and 30% respectively—in the FAM, ensuring that its properties meet all the requirements of the relevant standards and guaranteeing resistance to low-temperature cracking.

## 1. Introduction

In light of climate change, particular attention is being placed on reducing the energy consumption of technological processes involved in the production of materials. Therefore, a priority in road construction is the implementation of technologies for producing materials with reduced energy requirements, which are consequently environmentally friendly due to lower greenhouse gas emissions, particularly CO_2_ [1,2,3]. In road construction, one of the most energy-intensive technological processes is the production of asphalt mixtures using ‘hot mix asphalt’ (HMA) technology. These are produced at high temperatures ranging from 160 °C to 180 °C. Lowering the production temperature, therefore, reduces the energy consumption of the production process [4,5]. As a result, an additional beneficial effect is achieved in the form of reduced bitumen ageing, which has a significant impact on the durability of the asphalt pavement [6,7]. In order to lower the production temperature of the asphalt mixture, it is necessary to reduce the viscosity of the asphalt binder during the production process without having to heat it to a high temperature. To achieve this, low-viscosity additives [8,9], surfactants [10,11] and various types of chemicals [12,13,14] are added to the asphalt binder.

Another significant achievement is the reduction in CO_2_ emissions and other volatile compounds released during the asphalt mixture production process, which plays a significant role in mitigating the ‘greenhouse’ effect [1]. Furthermore, as a result of the smaller temperature gradient between this type of asphalt mixture and the ambient temperature, the cooling process is slowed down, which helps to extend the transport range from the production plant to the point where it is incorporated into the structural layer of the pavement surface.

The first stage in the process of introducing this type of asphalt mixture into global road construction practice was the development of WMA (Warm Mix Asphalt) technology, which is characterised by a reduction in the production temperature of approximately 30 °C to 40 °C compared to traditional asphalt mixtures [2,4]. Research findings have shown that every 10 °C reduction in production temperature results in savings of approximately 1.0 L of fuel and 1.0 kg of CO_2_ emissions per tonne of asphalt mixture produced [1,3].

However, it is the foamed mixture asphalt (FMA) that is the most energy-efficient material, as its production temperature is reduced by between 45 °C and 60 °C compared to the production temperatures of HMA [15,16]. In order to ensure that the FMA mixture possesses the required properties comparable to those of HMA, particular attention must be paid to the quality of the foamed asphalt binder used [17,18]. The development of foamed asphalt binder technology began at the end of the 20th century [19], and its dynamic development occurred at the beginning of the 21st century [20], when it became widely used in the cold recycling process for pavement rehabilitation [21,22,23,24,25,26,27,28]. Currently, however, the latest research focuses on the implementation of foamed asphalt binder in FAM mixtures [29,30,31,32,33] intended for the upper layers of asphalt pavement structures. These studies focus on obtaining foamed asphalt binder with the most favourable foaming parameters, namely maximum expansion (ER) and half-life of the foamed asphalt binder (HL_a_). To this end, the effect of various additives dosed into the asphalt binder prior to its foaming with water was investigated [34,35]. The most commonly used additive was Fischer–Tropsch (F-T) synthetic wax, which reduces the viscosity of the asphalt binder and improves its basic and rheological properties [36,37] as well as the properties of the asphalt mixture [38]. Unfortunately, when the temperature at which the FAM is incorporated into the pavement structure is reduced below 90 °C, synthetic wax crystals form [39], making compaction more difficult. For this reason, in addition to F-T synthetic wax, various other chemical additives have been considered, including surface-active agents (SAAs) [11], which significantly reduce the viscosity of the binder, resulting in a marked improvement in the foaming characteristics of the asphalt [11]. A further advantage is their significantly lower cost of use compared with synthetic wax. Unfortunately, however, a certain adverse effect of SAAs on the standard properties of asphalt binder has been observed and consequently on the asphalt mixture, which may fail to achieve the required properties [40]. In such cases, hydrated lime is used in HMA technology as a partial substitute for limestone filler [41,42,43]. It has a beneficial effect on ensuring the asphalt mixture’s resistance to moisture and frost, as well as on the mechanical characteristics of asphalt concrete, such as the stiffness modulus and resistance to permanent deformation [44,45]. Furthermore, hydrated lime also slows down the ageing process of the asphalt binder, which has a beneficial effect on the service life of the asphalt pavement [46,47].

Consequently, when developing the FMA, an SAA was selected as an additive to be added to the asphalt binder prior to foaming, and hydrated lime was selected as a partial substitute for the mineral filler. The developed FMA should possess both the required basic properties, such as those of a traditional asphalt mixture [48], and resistance to low-temperature cracking. These properties are particularly important in countries with a temperate climate, where sub-zero temperatures affect the asphalt pavement.

## 2. Materials and Methods

### 2.1. Materials

#### 2.1.1. Asphalt Binder

The study used 50/70 paving asphalt binder produced by the ORLEN S. A. refinery in Płock (Poland). It is the primary binder used in asphalt mixtures in Central and Eastern Europe [48].

Prior to foaming, the 50/70 asphalt binder was modified with 0.6% by weight (relative to the 50/70 asphalt binder) of a surface-active agent (SAA), which is produced on the basis of a fatty acid amide. Detailed results for the 50/70 bitumen with the SAA are presented in [11].

The asphalt binder with the SAA additive was foamed with water in a Wirtgen WLB-10S foaming (Wirtgen GmbH, Windhagen, Germany) unit to determine the maximum expansion ratio (ER) [20,49] and the half-life of the asphalt binder foam (HL_a_) [18,49]. The foaming parameters of the modified 50/70 asphalt binder were determined by dosing foaming water (FWC—Foaming Water Content) during the foaming process at the following levels: 1.5% by weight, 2.0% by weight, 2.5% by weight, 3.0% by weight, 3.5% by weight and 4.0% by weight, in accordance with [11,49].

**Figure 1:**
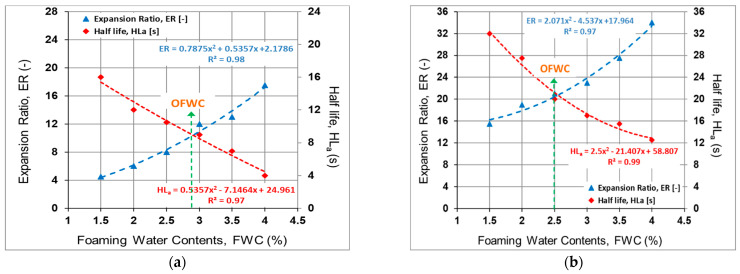
Foaming characteristics: (**a**) 50/70 asphalt binder; (**b**) 50/70 asphalt binder with 0.6% SAA.

The results for selected properties of unmodified 50/70 asphalt binder and 0.6% SAA-modified asphalt binder are summarised in Table 1 [11]. The foaming characteristics of the asphalt binder before and after modification are shown in Figure 1.

The addition of 0.6% SAA to the 50/70 asphalt binder prior to its foaming with water resulted in an almost twofold increase in the ER and HL_a_ foaming performances. Consequently, the production of FMA will be significantly more efficient than when unmodified foamed asphalt binder was used.

#### 2.1.2. Hydrated Lime

In accordance with the experimental design, hydrated lime that met the requirements of EN 459-1 [55] was used in the study.

In the production of the asphalt mixture, hydrated lime replaced part of the limestone powder at levels of 15%, 30% and 45% by weight. It was added to the limestone powder, then mixed to achieve homogeneity and sieved through a 100 μm sieve. The homogeneity of the new mineral powder was assessed macroscopically. Particular care was taken to ensure that no lumps of hydrated lime were present in the newly produced mineral powder. Only then was the newly produced mineral powder added to the remaining mineral mixture, to which the 50/70 foamed bitumen with 0.6 SAA was added using a WLB-10S unit, in order to produce an asphalt mixture at a temperature of 120 °C.

#### 2.1.3. Mineral Mix Design

In order to assess the effect of hydrated lime on the FAM with the modified foamed asphalt binder containing SAA, an AC 8 S asphalt mixture intended for the wearing course of the pavement was used, in which 5.6% binder was employed in accordance with the relevant requirements [48]. In connection with the use of hydrated lime in the FAM, and in order to determine the optimum binder content—given the need to account for the increased binder demand of the asphalt mixture —for reference purposes, it was also used in absolute quantities of 5.9%, 6.2% and 6.5%.

The mineral mix composition of the foamed asphalt mixture AC 8 S was as follows:

- Filler (limestone): 7.0%;
- Crushed fine continuously graded aggregate 0/2 mm (limestone): 37.0%;
- Coarse aggregate 2/5 mm (gabbro): 16%;
- Coarse aggregate 4/8 mm (gabbro): 40%.

The aggregates used in the mineral mixture met the requirements set out in WT-2 2014 [48].

**Figure 2:**
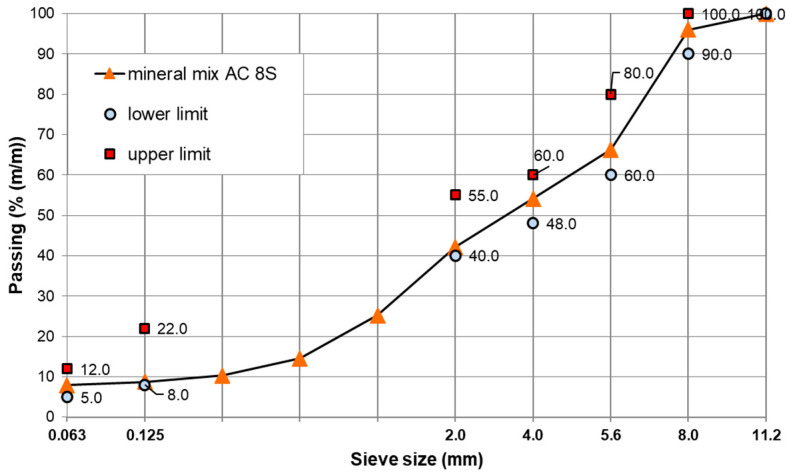
Graining curve of the foam mix asphalt AC 8 S mineral mixture with limit points [48].

Basic compositions of the FMA mineral mix are shown in Figure 2.

The properties of the reference FMA AC 8 S mixture (5.6% of asphalt binder 50/70 [11]) are listed in Table 2.

#### 2.1.4. FAM Production Procedure

The FAM AC 8 S mix was prepared in a heated 60-litre mechanical mixer, fitted with a temperature control system accurate to 1 °C. First, the mineral components were fed into the mixer, followed by the foamed asphalt binder with the 0.6 SAA additive, produced in the WLB-10S plant. In accordance with the test plan, the study utilised varying amounts of modified foamed asphalt binder, ranging from 5.6% to 6.5%, in order to assess its effect on the analysed properties of the FAM. Consequently, the gradation of the aggregate mixture was adjusted to the amount of foamed asphalt binder dosed. To ensure the required values of the asphalt concrete parameters, in accordance with the experimental plan, hydrated lime was dosed in quantities of 15%, 30% and 45% by weight to replace an equivalent amount of mineral filler. The asphalt mixture was mechanically mixed using a mixer at a speed of 30 revolutions per minute for a period of 5 min. The production temperature of AC 8 S with additives did not exceed 120 °C.

The mixture prepared in this way was compacted using a Marshall impact compactor. The number of blows depended on the type of test to which it was subjected. After the asphalt mixture sample was prepared, it was left to cool at room temperature for 48 h to allow any residual water from the asphalt foaming process to evaporate and to ensure proper adhesion of the binder to the mineral aggregate.

### 2.2. Test Methods

The main objective of this study was to determine the effect of hydrated lime on the mechanical and physical properties, and in particular on the resistance to low temperatures, of a mixture produced using the FMA process with a foamed asphalt binder with 0.6% SAA. The following parameters of the AC 8 S mixture were taken into account during the tests:

- Air void content (*V_a_*, %) as per EN 12697-8 [57];
- Resistance to moisture and frost (*TSR*, %) as per modified AASHTO T283 [59];
- Resistance to permanent deformation (*WTS_AIR_*, *PRD_AIR_*) as per EN 12697-22 [60] and WT-2 2014 [48];
- Complex modulus in the 4PB-PR test (E*) as per EN 12697-26 [61];
- Resistance to low-temperature cracking (*R*_−2°C_, *MPa*) as per PANK 4308 [62];
- Resistance to crack propagation, SCB (ε_max_; σ_max_; K_lc_) as per EN 12697-44 [63];
- Resistance to low-temperature cracking testing, TSRST (σ_cry,_ T_failure_) as per EN 12697-46 [64].

The parameters V_a_, TSR, E* and R_−2°C_ were determined on samples compacted using the Marshall method, applying the number of blows specified for each procedure used. The parameters WTS_AIR_ and PRD_AIR_ were determined on slabs produced in accordance with EN 12697-22 [60]. In contrast, the characteristics ε_max_, σ_max_, K_lc_, σ_cry_ and T_failure_ were determined on beams cut from FMA slabs prepared in accordance with the requirements [63,64]. The FAM samples met the specified requirements in terms of physical and geometric properties.

During the research process, attention was paid to finding the best compromise for the composition of the asphalt mixture produced using FAM technology, in terms of the content of modified foamed bitumen and hydrated lime as a partial substitute for mineral powder. The foamed bitumen was optimised in terms of the foaming parameters ER and HL_a_ [11].

The research methods presented here relate solely to those properties for which the test results were used in the process of optimising the properties of the asphalt mixture. The number of samples used in the tests depended on the requirements of the relevant standards but could not be less than the number of estimated model parameters [65]. Consequently, the number of samples ranged from 3 to 6 for each combination of hydrated lime and modified foamed asphalt binder included in the experimental design.

#### 2.2.1. Air Void Content

The void content (Va) is an important characteristic of an asphalt mixture, which determines its other properties. It was determined in accordance with standard EN 12697-8 [57] using Equation (1):

(1) $$V_{a}=\frac{\rho_{m}-\rho_{b}}{\rho_{m}}\cdot 100\%$$

where

V_a_ is the air void content (0.1%);

ρ_m_ is the asphalt mixture density (Mg/m^3^);

ρ_b_ is the asphalt mixture bulk density (Mg/m^3^).

The density of the foamed asphalt mixture was determined with a pycnometer following procedure A (ρ_mv_) according to EN 12697-5:2010 [66]. The bulk density was determined following procedure A (ρ_bdry_) according to EN 12697-6:2012 [67].

#### 2.2.2. Resistance of Asphalt Mixture to the Effects of Water and Frost in Accordance with AASHTO T283

The FAM AC 8 S water and frost resistance tests were carried out on Marshall specimens (63.5 mm ± 2.5 mm in height and 101 mm in diameter, with a void content of between 6% and 8%). The specimens were conditioned in accordance with a procedure involving one freeze-thaw cycle [59].

The TSR water and frost resistance index, in accordance with AASHTO T283 [61], was calculated using Formula (2):

(2) $$TSR=\frac{{ITS}_{w}^{A}}{{ITS}_{d}^{A}}\cdot 100\%$$

where

ITS_d_ ^A^ is the average indirect tensile strength of specimens conditioned in air;

ITS_w_ ^A^ is the average indirect tensile strength of specimens conditioned in water with one freeze-thaw cycle in accordance with AASHTO T283.

#### 2.2.3. Permanent Deformation

Resistance to permanent deformation of the asphalt mixture was assessed according to EN 12697-22 [60] and WT-2 2014 [48] at a temperature of 60 °C. Results were used to determine the slope of the WTS_AIR_ rutting plot and the proportional rut depth (*PRD_AIR_*). The proportional rut depth and the maximum rut depth gain were calculated using Equations (3) and (4):

(3) $${PRD}_{AIR}=\frac{{RD}_{AIR}}{h}\cdot 100\%,$$

(4) $${WTS}_{AIR}=\frac{\left(d_{10000}-d_{5000}\right)}{5}[mm/10000cycles]$$

where

RD_AIR_ is the rut depth (mm);

d_10000_ is the rut depth after 10,000 cycles (mm);

d_5000_ is the rut depth after 5000 cycles (mm);

h is the specimen height (mm).

For further analysis, the arithmetic mean of the five tests performed in each test series was used as the final result of the FAM AC 8 S resistance to permanent deformation.

#### 2.2.4. Complex Modulus of Stiffness in the 4PB-PR Test

**Figure 3:**
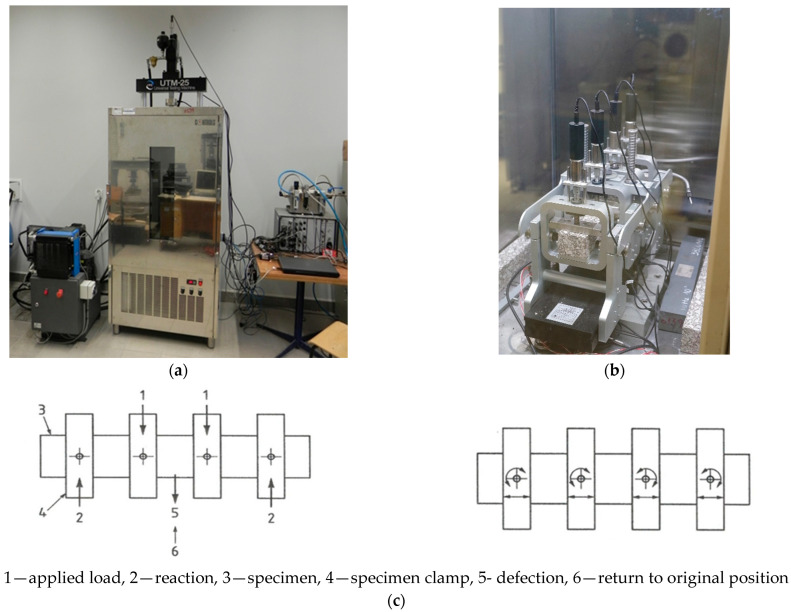
Test frame and 4PB-PR specimen: (**a**) thermoregulation chamber (M.M. Iwański); (**b**) 4PB-PR specimen during the test for the complex modulus of stiffness (M.M. Iwański); (**c**) load diagram in the 4PB-PR tests (EN 12697-26) [61].

The composite stiffness module in the 4PB-PR four-point bending beam diagram was determined in accordance with the load diagram in the 4PB-PR test (EN 12697-26) [61]. The test was carried out on a compacted cuboid specimen under a sinusoidal load. The bending process is induced by the movement of the central points, which apply a vertical load to the material, perpendicular to the longitudinal axis. The periodic displacement should be symmetrical about the zero point and sinusoidal, whilst the amplitude of the displacement should remain constant. It is recommended that, during the test, the force required to induce deformation of the specimen be measured as a function of time, together with the phase angle between the force pulse and the displacement. The test was carried out at a temperature of 10 °C and a frequency of 10 Hz. The frequency of 10 Hz corresponds to the highest speed of heavy goods vehicles considered in the test, within the range of 65 km/h to 70 km/h. The temperature-control unit and the FMA specimen mounted on the 4PB-PR beam are shown in Figure 3.

#### 2.2.5. Resistance to Low-Temperature Cracking According to PANK 4302 Method

The weather resistance was determined based on the Finnish standard PANK 4302 [62]. The value of the failure force was determined following an indirect tension protocol at the test temperature of −2 °C (±1 °C). Test specimens were prepared with a Marshall hammer using 75 blows per specimen face (2 × 75). After 28 days of air-dry curing, the samples were conditioned for 16 h at −2 °C, followed directly by destructive testing. Weather resistance was calculated from Formula (5):

(5) $$R_{-2{}^{\circ}C}=\frac{2*P}{\pi *h*d}$$

where

R_−2°C_ is the indirect tensile strength, an indicator of the resistance to weather and climate factors;

P is the maximum failure force (kN);

h is the height of the specimen, rounded off to the nearest 0.1 mm (mm);

d is the diameter of the specimen, rounded off to the nearest 0.1 mm (mm).

In accordance with the requirements of the standard, it is assumed that if the R_−2°C_ parameter is less than 4.8 MPa, the asphalt mixture is resistant to the effects of water and to the formation of cracks at low temperatures.

#### 2.2.6. Crack Propagation in the SCB Test

Another very important characteristic of FAM AC 8 S was the assessment of the asphalt mixture’s resistance to crack propagation, which was evaluated whilst the specimen was being bent in accordance with the SCB procedure [63].

**Figure 4:**
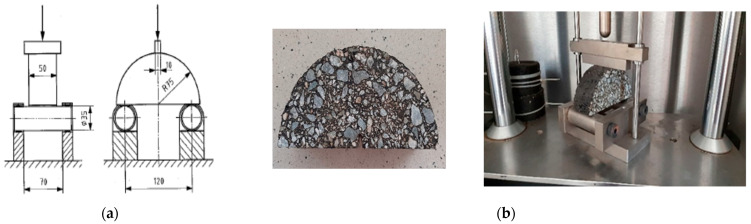
Test frame and SCB specimen: (**a**) schematic diagram (EN 12697-44) [63]; (**b**) SCB specimen during the test for the resistance to cracking (M.M. Iwański).

The recommended formulations of AC 8 S were used in the test. The SCB specimens of AC 8 S had a notch with a depth of 10.3 mm and a width of 1 mm. Each specimen was subjected to a three-point bending test. The centre of the specimen base was subjected to tensile stress. During the test, the strain was increased at a constant rate of 5 mm/min. The corresponding pressure increased to the maximum value F_max_, which is directly related to the cracking resistance of the specimen. Figure 4 shows the test frame and the specimen.

The specimens of AC 8 S were prepared according to the requirements of EN 12697-31 [68]. The following quantities were determined:

- Strain at maximum force, *ε*_max_, from Formula (6):

(6) $$\varepsilon_{max,i}=\frac{\Delta W_{i}}{W_{i}}\times \;100\%$$

where *W*_i_ is the height of specimen i (i = 1, 2, 3, 4) (mm);

Δ*W*_i_ is the vertical strain at maximum force for specimen i (i = 1, 2, 3, 4) (mm).

- Maximum stress at damage, *σ*_max,i_, from Formula (7):

(7) $$\sigma_{max,i}=\frac{F_{max,i}}{D_{i}t_{i}}\;(N/{mm}^{2})$$

where *D*_i_ is the diameter of specimen i (i = 1, 2, 3, 4) (mm);

*t*_i_ is the thickness of specimen i (i = 1, 2, 3, 4) (mm);

*F*_max,i_ is the maximum force for i (i = 1, 2, 3, 4) (Newton).

- Resistance to cracking, *K*_Ic_, specimen *i* (i = 1, 2, 3, 4) from Formula (8):

(8) $$K_{Ic,i}=\sigma_{max,i}\cdot Y\cdot \sqrt{\pi \cdot a_{1}}\;\;(N/{mm}^{3/2})$$

where *a*_i_ is the crack depth of specimen i (i = 1, 2, 3, 4) (mm);

σ_max,i_ is the peak stress for specimen i (i = 1, 2, 3, 4) (N/mm^2^).

*Y* is the normalized yield stress according to Formula (9):

(9) $$Y=4.782-1.219\cdot \left(\frac{a_{i}}{r_{i}}\right)+0.063exp\left(7.045\cdot \left(\frac{a_{i}}{r_{i}}\right)\right)$$

where

r_i_ is the radius of specimen i (i = 1, 2, 3, 4) (mm).

The specimens that were 150 ± 1 mm in diameter were molded in a gyratory compactor and compacted in accordance with the requirements of EN 12697-31 [68].

The specimen thickness was 50 ± 3 mm. When sawing the specimens from the compacted samples, care was taken to ensure that upper and lower surfaces were flat and parallel, with trimming as necessary.

The notch N cut in the specimen central part was 1.0 ± 0.10 mm in nominal width and (10.0 ± 1.0 mm) in depth. The specimens were tested at 0 °C. Prior to the tests, the specimens were placed in a thermostatic chamber at 0 ± 1 °C for at least 4 h.

#### 2.2.7. Low-Temperate Cracking and Properties by Uniaxial Tension Tests

The assessment of the influence of hydrated lime on the rheological properties—namely, σ_cry_ (the maximum thermally induced stress at specimen fracture) and T_failure_ (the fracture temperature)—of the FMA AC 8 S mixture was carried out in accordance with EN 12697-46 [64].

**Figure 5:**
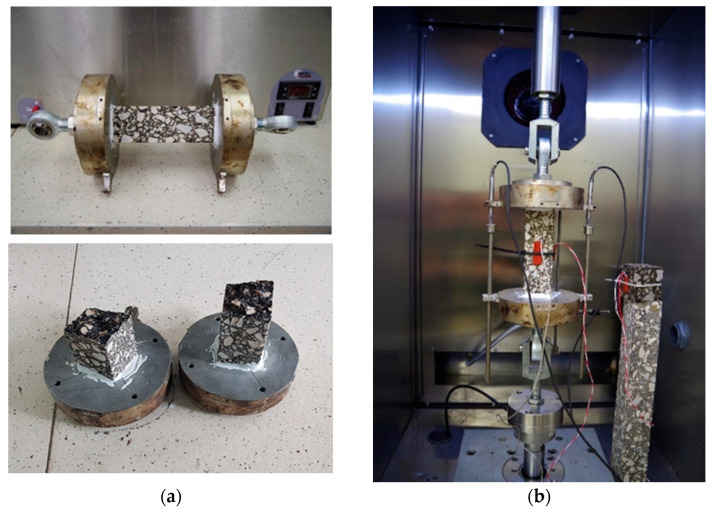
TSRST low-temperature cracking testing (EN 12697-46 [64]): (**a**) TSRST specimen before and after testing (M.M. Iwański); (**b**) specimen during TSRST characteristics testing (M.M. Iwański).

The test was carried out on specimens measuring 50 × 50 × 250 mm. Steel discs were affixed to the top and bottom faces of the specimen to enable it to be secured in the frame of the UTM-12 machine (CONTROLS S.p.A., Liscate, Italy). Extensometers were attached to the three side faces of the specimen to measure its deformation. A temperature sensor was positioned on the fourth face of the specimen. The specimen, prepared in this way, was placed inside the UTM-12 thermostatic chamber. A view of the specimen prepared for testing and the entire UTM-12 test rig is shown in Figure 5.

### 2.3. Design of the Experiment

The study utilised two variables: hydrated lime and a foamed asphalt binder with 0.6% SAA contained in FMA AC 8 S. Consequently, the experimental design was based on the principles of the factorial experimental design algorithm [69]. The properties of the foamed asphalt mixture AC 8 S were investigated within the scope of a 4 × 4 factorial design, in accordance with the adopted research programme. The foamed asphalt binder modified with the SAA was added at levels of 5.6%, 5.9%, 6.2% and 6.5% to the FMA. Hydrated lime, on the other hand, was added to replace part of the limestone powder at levels of 0%, 15%, 30% and 45% by weight during the production of FAM AC 8 S.

The test results were subjected to statistical analysis, the aim of which was to determine the significance of the effect of hydrated lime (HL) and the foamed asphalt binder with 0.6% SAA (FA) on the properties of the FMA using analysis of variance (ANOVA) [69].

The FA and HL values were identified as significant factors influencing the analysed property of the foamed asphalt mixture when the *p*-value was lower than the accepted significance level of α = 0.5.

In order to provide a comprehensive description of the variation in the analysed property (A) of the FMA AC 8 S mixture in terms of the content of the 50/70 foamed asphalt binder with 0.6% SAA (FA) and hydrated lime (HL), a statistical model utilising a second-order polynomial [56,60] was adopted, in accordance with Formula (10):

(10) $$A=b_{0}+b_{1}\cdot x_{1}+b_{2}\cdot x_{2}+\;b_{3}\cdot x_{1}\cdot x_{2}+b_{4}\cdot x_{1}^{2}+\;b_{5}\cdot x_{2}^{2}$$

where x_1_ is the foamed asphalt binder with the 0.6% SAA—FA (%);

x_2_ is the hydrated lime—HL (%);

b_0_–b_5_ is regression coefficients.

In order to assess the impact of the FA and HL effects on the properties of FMA AC 8 S, as well as the interaction between them, a Pareto analysis was used [70]. The significance of the FA and HL effects was assessed, and the intensity of their impact on the analysed model parameter and the direction of the trend were determined. The values on the Pareto chart corresponded to standardised relative effect scores.

In order to determine the optimum quantities of HL and FA in foamed asphalt mixture AC 8 S, multi-criteria statistical optimisation using a generalised objective function was employed [71,72]. The results obtained during the optimisation process were visualised using the statistical software Statistica 13.3 [73]. The most desirable values for a given property are assigned a value of ‘1’, whilst a value of ‘0’ corresponds to unacceptable values for that material property [71,72]. However, when a large number of parameters are being analysed and correlations may exist between them, it is advisable to combine these separate values into a desirability index (D). It is then possible to make comparisons and determine optimal relationships between the analysed characteristics. The desirability index D is calculated according to Equation (11):

(11) $$D={\left[\prod_{u=1}^{n}d_{u}\right]}^{\frac{1}{n}}$$

where

n is the number of variables;

d_u_ is the value of the individual desirability.

## 3. Results and Discussion

### 3.1. Air Void Content in FAM AC 8 S with Foamed Asphalt Binder and Hydrated Lime

The key parameter of the foamed asphalt mixture AC 8 S that was analysed was the void content (Va). This essentially determines the quality of the FAM in terms of the other parameters. The void content (Va) of the foamed asphalt mixture should be within the range of 2.0% to 4.5%, in accordance with WT-2 2014 [48]. The tests were conducted on five samples from each batch of asphalt mixture containing different binder and hydrated lime contents. The standard deviation in each test batch was no greater than 0.5%, and the coefficient of variation was no greater than 12%.

The effects of FA and HL on the void content (Va) of foamed mix asphalt (FMA) were determined on the basis of a regression model in accordance with (10) and using the assumptions set out in [70]. The significance of the effects of FA and HL and the effect of their interaction on V_a_ was determined using analysis of variance (ANOVA). The parameters of the regression model predicting the void content (V_a_) of foamed asphalt mixture, together with the optimisation quality parameters, are presented in Table 3.

**Figure 6:**
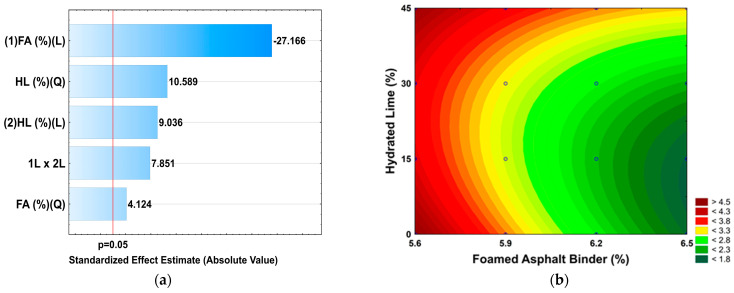
Air void content V_a_ of foamed asphalt mixture AC 8 S as a function of the quantity of FA and HL content: (**a**) Pareto diagram; (**b**) response surface.

By contrast, the analysis of the significance of the effects of FA and HL, as well as the interaction between them, on the variability of the V_a_ property of the foamed asphalt mixture is presented graphically using the relationships shown in the Pareto chart (Figure 6a). The regression model developed, in the form of a response surface illustrating the relationship between the FA and HL additives and the V_a_ variable of the foamed asphalt mixture, is shown in Figure 6b.

An analysis of the relationship shown in Figure 6a reveals that the content of the foamed asphalt binder with 0.6% SAA and hydrated lime has the greatest influence on the variability of Va in the foamed asphalt mixture AC 8 S. However, the trend for the influence of FA shows a decreasing trend, indicating that increasing the amount of binder does not have a significant effect on Va. In contrast, the influence of hydrated lime is characterised by an increasing trend; thus, an increase in the concentration of HL has a beneficial effect on Va. An analysis of the results presented in the Pareto chart shows that the effects of FA and HL on the FMA are not only highly significant, but that there was also a beneficial interaction between them, which influenced the Va characteristics. Thus, there is a synergy between FA and HL, which ensures their beneficial effect on Va.

Increasing the FA and HL content in FAM AC 8 S results in a reduction in the void content across the entire experimental range. Analysis of the test results indicates that the effect of hydrated lime on Va is particularly significant in the FA range from 5.6% to 5.9%. The increase in the void ratio is significant and exceeds the recommended maximum of 4.0%. Increasing the FA content from 5.9% to 6.2%, with an HL content of 15–30% in the FAM, allows this parameter to achieve the most favourable void ratio V_a_, in accordance with [48]. A further increase in the FA content to 6.5% results in a noticeable reduction in the void content Va in the FAM, falling below the recommended lower limit in accordance with WT-2 2014 [48]. To summarise, it should be noted that the effect of hydrated lime on the void content V_a_ of the FAM is consistent with that observed for a traditional asphalt mixture; it is the result of increased adhesion of the asphalt binder with 0.6% SAA to the aggregate [74,75].

### 3.2. Resistance to Moisture and Frost of FAM AC 8 S Using Foamed Asphalt Binder with 0.6% SAA and Hydrated Lime According to AASHTO T283

In accordance with the requirements of AASHTO T283, an asphalt mixture is considered to be resistant to moisture and frost when its TSR value reaches at least 80%. This property is particularly important when constructing asphalt pavements in countries with a temperate and humid climate, as it determines their service life.

The effect of the content of foamed asphalt binder with 0.6% SAA and hydrated lime on the moisture and frost resistance of the foamed asphalt mixture was determined in accordance with the established methodology presented during the analysis of the void content (V_a_). The tests were conducted on five samples from each batch of the asphalt mixture containing different binder and hydrated lime contents. The standard deviation in each test batch was no greater than 6.2%, and the coefficient of variation was no greater than 9%.

**Figure 7:**
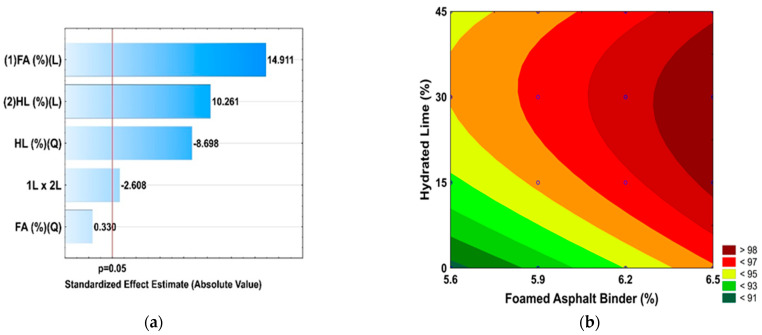
Resistance to moisture and frost TSR (AASHTO T283) of foamed asphalt mixture AC 8 S as a function of the quantity of FA and HL content: (**a**) Pareto diagram; (**b**) response surface.

A regression model relating TSR to FA and HL was developed in accordance with (10), and the intensity of their interaction was determined [72] and is shown in Table 4. The results of the analyses are presented graphically in the form of a Pareto chart in Figure 7a, and the parameters of the TSR regression model are shown in Figure 7b.

An analysis of the relationships shown in the Pareto chart (Figure 7a) indicates that FA and HL have a very significant impact on the TSR index, ensuring it remains at a high level. This will therefore guarantee that the foamed asphalt mixture is highly resistant to the effects of moisture and frost. Hydrated lime improves the adhesion of the binder to the aggregate, resulting in increased resistance to moisture and frost, as also confirmed by test results for the use of hydrated lime in traditional asphalt mixtures [76,77]. Furthermore, there is also a synergy between FA and HL, which plays a significant role in ensuring the moisture and frost resistance of the foamed asphalt mixture AC 8 S, although the trend in this regard is decreasing, which is unfavourable. This phenomenon may stem from the fact that, if the optimum amount of hydrated lime in an asphalt mixture is exceeded, there is an increase in the volume of voids, which reduces its resistance to the effects of moisture and frost.

It can be concluded that as the binder content in FAM AC 8 S increases, the TSR value, i.e., resistance to moisture and frost, also increases. This behaviour of the TSR parameter was expected and is consistent with the general principles of asphalt materials technology. The use of hydrated lime ensures favourable values for the TSR parameter under analysis, and with a 30% HL content in the mineral powder and a modified foamed asphalt binder with 0.6% SAA content of over 5.9% in the FAM, a high level of moisture and frost resistance for the asphalt pavement—at least 90%—is guaranteed.

### 3.3. Resistance to Permanent Deformation of FAM AC 8 S

Permanent deformation resistance of the AC 8 S asphalt mixture containing the recommended 5.6%, 5.9%, 6.2% and 6.5% of the 50/70 foamed asphalt binder with 0.6% SAA and hydrated lime at concentrations of 0%, 15%, 30% and 45% was assessed in accordance with the requirements set out in WT-2 2014 [48]. The tests were conducted on five samples from each batch of the asphalt mixture containing different binder and hydrated lime contents. The tests were conducted on five samples from each batch of the asphalt mixture with different binder and hydrated lime contents. The standard deviation in each batch for WTS_AIR_ and PRD_AIR_ did not exceed 0.015 mm/10^3^ cycles and 0.2%, respectively, and the coefficients of variation did not exceed 11.9% and 1.7%, respectively. FAM AC 8 S should exhibit the following resistance values: WTS_AIR_ < 0.15 mm/10^3^ cycles and PRD_AIR_ < 9% [48].

The parameters of the regression model predicting WTS_AIR_ and PRD_AIR_ FAM, together with the goodness-of-fit parameters, are presented in Table 5.

An analysis of the parameters presented in Table 5 shows that the quantities of the foamed asphalt binder and hydrated lime were significant factors influencing the resistance to permanent deformation of FMA AC 8 S, as characterised by the WTS_AIR_ and PRD_AIR_ parameters, as the associated *p*-values are lower than the adopted significance level of α = 0.05. Unfortunately, no synergistic effect between the additives (FA and HL) can be observed, which influences the trend in the change in the permanent deformation resistance of FAM AC 8 S. It should also be noted that the effects of FA and HL on PRD_AIR_ are more significant than on WTS_AIR_ of the FAM.

**Figure 8:**
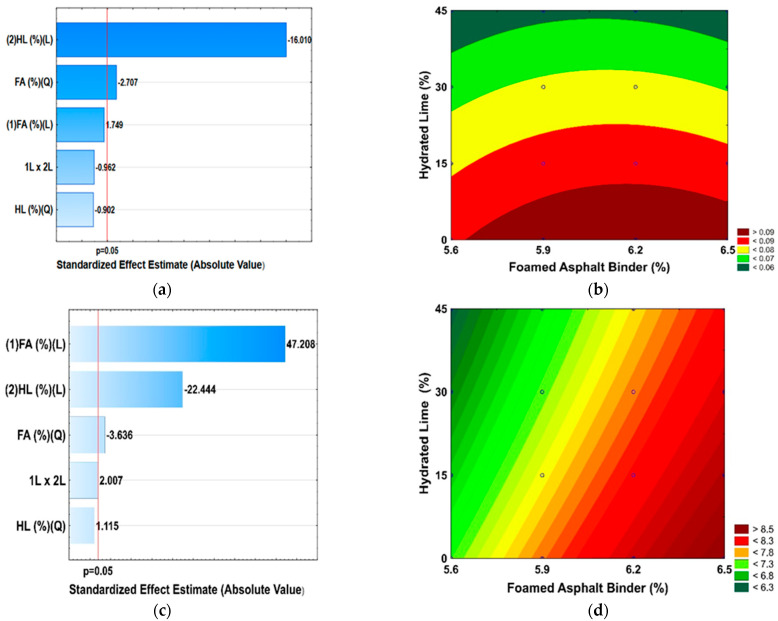
Resistance to permanent deformation of foamed asphalt mixture AC 8 S as a function of FA and HL lime content: (**a**) Pareto diagram of WTS_AIR_; (**b**) response surface of WTS_AIR_; (**c**) Pareto diagram of PRD_AIR_; (**d**) response surface of PRD_AIR_.

In addition, a detailed analysis of the significance of the effects of FA and HL, as well as the interaction between them, on the variability in FAM’s resistance to permanent deformation based on the WTS_AIR_ and PRD_AIR_ properties is presented in the form of a Pareto chart (Figure 8a,c). The regression model developed, in the form of a response surface illustrating the relationship between FA and HL and the WTS_AIR_ and PRD_AIR_ variables for FAM AC 8 S, is shown in Figure 8.

An analysis of the relationships shown in the Pareto chart (Figure 8a,c) confirms that foamed asphalt binder and hydrated lime have a very significant influence on the WTS_AIR_ and PRD_AIR_ indices, which characterise the resistance of FAM AC 8 S to permanent deformation. Unfortunately, the HL content shows a decreasing trend in the slope of the WTS_AIR_ rutting plot for the FAM. In contrast, the interaction between FA and HL has a more significant influence on the PRD_AIR_ characteristics. The trend in the influence of FA shows an upward trend, indicating its dominant role in determining the proportional rut depth (PRD_AIR_) of foamed asphalt mixtures. It can be concluded that foamed asphalt binder will counteract the stiffening effect of hydrated lime.

It can be concluded that, with a hydrated lime content of over 15% in the mineral powder and practically regardless of the amount of foamed asphalt binder containing 0.6% SAA, a high value for the slope of the WTS_AIR_ rutting plot (FAM) is ensured. In contrast, the proportional rut depth (PRD_AIR_) of foamed asphalt mixtures achieves the most favourable values when the content of modified foamed asphalt binder is in the range of 5.6% to 5.9%, regardless of the amount of hydrated lime added. An increase in the content of modified foamed asphalt binder above 5.9% has an adverse effect on the analysed PRD_AIR_ characteristic of the resistance to permanent deformation of FAM AC 8 S. This effect of the binder on the resistance to permanent deformation of the FAM is consistent with the results of studies on traditional asphalt mixtures [40].

To summarise, the greatest positive influence on the WTS_AIR_ and PRD_AIR_ values, which characterise the resistance to permanent deformation of FAM AC 8 S, is exerted by the content of hydrated lime. Increasing the concentration of hydrated lime has a positive effect on the WTS_AIR_ and PRD_AIR_ values. Conversely, increasing the amount of foamed asphalt binder with 0.6% SAA shows the opposite trend, which is consistent with the behaviour of the binder in traditional asphalt mixtures [40]. FMA AC 8 S, containing at least 15% hydrated lime in place of limestone powder and up to 5.9% modified foamed asphalt binder, exhibits the most favourable permanent deformation resistance parameters (WTS_AIR_ and PRD_AIR_).

### 3.4. Resistance to Low-Temperature Cracking

Low-temperature resistance tests of the FAM AC 8 S were conducted on five samples from each batch of the asphalt mixture with different binder and hydrated lime contents. The standard deviation in each test batch did not exceed 0.39 MPa, and the coefficient of variation did not exceed 12.9%.

The parameters of the regression model predicting the low-temperature resistance of the FAM AC 8 S, based on a test at −2 °C [62], together with the fit quality parameters (HL, FA) derived using ANOVA analysis, are presented in Table 6.

**Figure 9:**
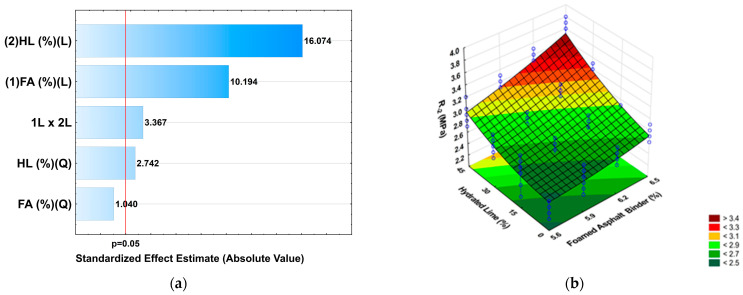
Resistance of FMA AC 8 S to conditions according in −2 °C as a function of FA and HL content: (**a**) Pareto diagram; (**b**) response surface.

In contrast, the analysis of the qualitative influence of FA and HL, as well as the interaction between them, on the variability of the R_−2_ property of FMA AC 8 S, presented graphically using the STATISTICA [73], is presented in the form of a Pareto chart (Figure 9a). The regression model developed, in the form of a response surface illustrating the relationship between the additives (FA and HL) contained in foamed asphalt mixture AC 8 S and the R_−2_ variable (low-temperature resistance), is shown in Figure 9b.

Analysis of the Pareto chart shows that hydrated lime had the greatest positive effect on the change in the low-temperature resistance of FAM AC 8 S, as characterised by the R_−2_ parameter. To a lesser extent, though still significant, a strong positive effect is observed for foamed asphalt binder. It should also be noted that there is an interaction between the effects of hydrated lime and foamed asphalt binder with 0.6% SAA on the low-temperature resistance characteristics—parameter R_−2_—which should be regarded as a positive effect. It can be concluded that this positive effect on FMA low-temperature resistance stems from the use of hydrated lime, which ensures a high level of adhesion between the binder and the aggregate [78], and foamed asphalt binder, which ensures the appropriate flexibility of FMA.

It can be concluded that, across the entire scope of the experiment, FMA AC 8 S meets the specified requirements, namely that its indirect tensile strength is less than 4.8 MPa [62]. Consequently, a wearing course made from this material will not be susceptible to winter damage.

### 3.5. Complex Modulus of Stiffness (4PB-PR) for the Recommended Composition of FMA AC 8 S

The complex modulus of rigidity E* of an asphalt mixture is a very important parameter characterising its durability. It is therefore advisable to assess the effect of the additives used (HL and FA) on this characteristic of FAM AC 8 S. The tests were conducted on five samples from each batch of asphalt mixture containing different binder and hydrated lime contents. The standard deviation in each test batch was no greater than 460 MPa, and the coefficient of variation was no greater than 3.1%.

The parameters of the regression model predicting the complex modulus of stiffness E* in accordance with EN 12697-26 [61] for FAM AC 8 S at a temperature of 10 °C and a frequency of 10 Hz, together with the fit quality parameters (HL, FA) derived using ANNOVA analysis, are presented in Table 7.

**Figure 10:**
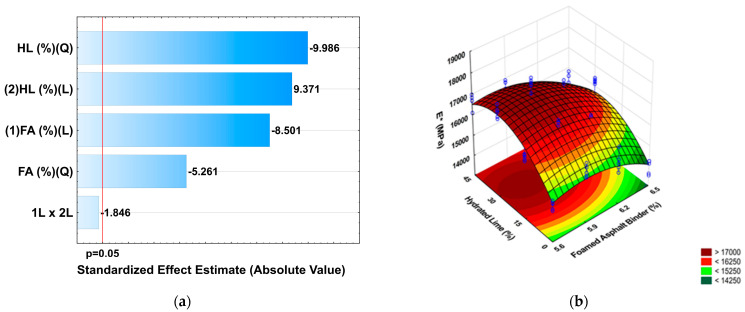
Complex modulus of stiffness (4PB-PR) of FMA AC 8 S as a function of FA and HL content: (**a**) Pareto diagram; (**b**) response surface.

An analysis of the significance of the qualitative effects of FA and HL used in the FAM, as well as the interaction between them on the variability of the E* trait, presented graphically using the STATISTICA [73], is presented using a Pareto chart (Figure 10a). The regression model developed in the form of a response surface illustrating the relationship between the composition of the FAM AC 8 S mixture and the E* variable is shown in Figure 10b.

Analysis of the Pareto chart shows that hydrated lime had the greatest positive impact on the change in the E* property of foamed asphalt mixtures. It can be concluded, however, that if its optimal value is exceeded, the stiffness of FAM will increase and this will have an adverse effect, as indicated by the HL (%) Q parameter on the Pareto chart. A lesser, though still significant, effect is also observed for foamed asphalt binder with 0.6% SAA. However, there is a downward trend, which indicates that increasing the amount of FA will have an adverse effect on E*. Unfortunately, no synergy is observed between HL and FA that would ensure a more favourable effect of the additives used on the E* FMA AC 8 S composite module. It can be observed that, in the case of both hydrated lime and foamed asphalt binder with SAA, a decreasing trend is evident, indicating that an increase in the content of hydrated lime and, in particular, foamed asphalt binder in FAM AC 8 S results in a reduction in the value of the complex modulus of elasticity E*.

The most favourable effect of the additives (FA and HL) on the complex modulus E* of foamed asphalt mixture AC 8 S is observed when the hydrated lime content in the aggregate exceeds 15% and when the content of foamed asphalt binder with 0.6% SAA is between 5.6% and 6.2%. The effect of lime on the complex modulus of rigidity E* of FAM AC 8 S is similar to that observed in a traditional asphalt mixture [79,80].

### 3.6. Crack Propagation (SCB) for the Recommended Composition of FMA AC 8 S

The crack propagation resistance characteristics in the SCB test in accordance with EN 12697-44 [63] for foamed asphalt mixture AC 8 S were determined by recording the force (F) and vertical deformation (∆Wi) as a function of time from the start of the test.

The analysis of the relationship between the parameters ε_max_, σ_max_ and K_lc_ and the amount of HL and FA used in the FAM was presented using regression models in accordance with Equation (10) and analysis of variance (ANOVA) [69]. The tests were conducted on five samples from each batch of asphalt mixture with different binder and hydrated lime contents. The standard deviation in each test batch for ε_max_, σ_max_ and K_lc_ did not exceed 0.035%, 0.08 N/mm^2^ and 2.08 N·mm^−3/2^, respectively, and the coefficient of variation did not exceed 4.2%, 6.8% and 7.1%, respectively. The fit of the model parameters to the experimental results for ε_max_, σ_max_ and K_lc_ is shown in Table 8.

**Figure 11:**
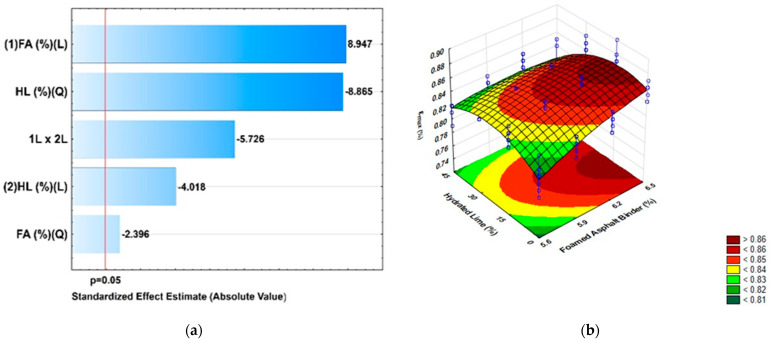

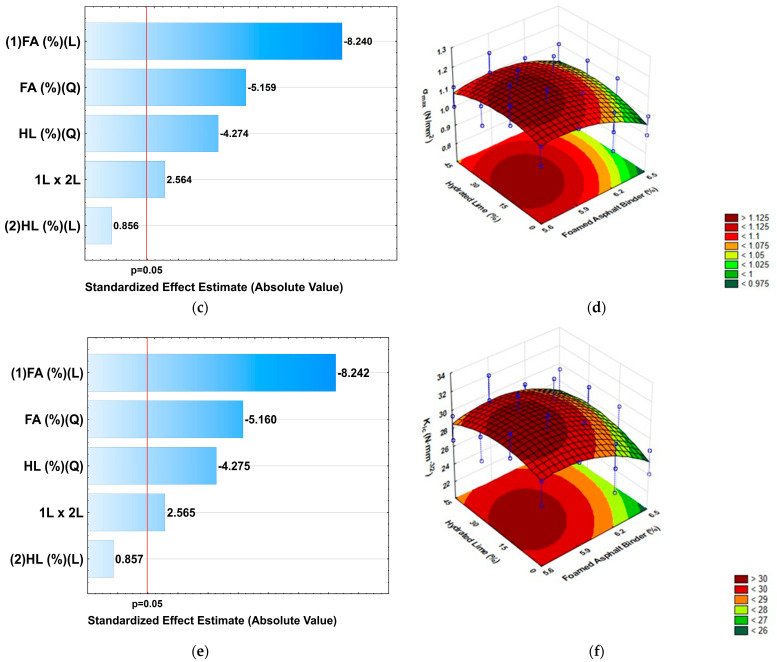
Characteristics of SCB of foamed asphalt mixture AC 8 S as a function of FA and HL content: (**a**) Pareto diagram of ε_max_; (**b**) ε_max_ response surface; (**c**) Pareto diagram of σ_max_; (**d**) σ_max_ response surface; (**e**) Pareto diagram of K_lc_; (**f**) K_lc_ response surface.

An analysis of the qualitative influence of foamed asphalt binder with 0.6% SAA and hydrated lime contained in FMA, as well as the interaction between them on the variability of crack propagation characteristics (ε_max_, σ_max_ and K_lc_) determined using the SCB, presented graphically using the STATISTICA software, is shown in the form of a Pareto chart (Figure 11). Meanwhile, the regression models developed for the analysed FAM characteristics, presented as surfaces, are shown in Figure 11.

Analysis of the Pareto chart shows that both foamed asphalt binder with 0.6% SAA and hydrated lime have the greatest impact on changes in the SCA characteristics (ε_max_, σ_max_ and K_lc_). In the case of ε_max_, the use of foamed bitumen in FMA AC 8 S shows an upward trend, indicating that increasing its quantity will have a significant effect on this parameter. No such relationship is observed for σ_max_ and K_lc_, which indicates that increasing the concentration of foamed bitumen will have an adverse effect on these characteristics. An adverse trend is observed in the effect of hydrated lime on the analysed SCB characteristics (ε_max_, σ_max_ and K_lc_). Increasing its quantity in FAM may result in a reduction in the values of the analysed characteristics. A beneficial effect of using modified foamed asphalt binder and hydrated lime is the occurrence of synergy between them, particularly with regard to the parameters σ_max_ and K_lc_. Thus, their effects will complement and reinforce one another, depending on the quantity of the additives used (FA and HL). Hydrated lime will stiffen the FAM, whilst modified foamed bitumen will ensure adequate ductility. Such a favourable synergy between FA and HL is not observed in the case of ε_max_, as there is an unfavourable decreasing trend.

Based on an analysis of the test results shown in Figure 11b, it can be concluded that the use of hydrated lime in quantities ranging from 15% to 30% and modified foamed asphalt binder in quantities exceeding 5.6% ensures a favourable value for the strain at maximum force (ε_max_) of FMA AC 8 S. Modified foamed asphalt binder will, to some extent, counteract the stiffening effect of hydrated lime and ensure high ε_max_ values.

An analysis of the test results for ultimate tensile strength (σ_max_) and crack resistance (K_lc_) highlights the particular role played by hydrated lime and modified foamed asphalt binder. With hydrated lime contents of 15% and 30% and modified foamed asphalt binder in the range of 5.6% to 5.9%, these parameters were at their highest levels. Thus, both additives influence the performance and ensure the required crack resistance of FAM AC 8 S.

To summarise, it can be concluded that the foamed asphalt mixture AC 8 S achieves its most favourable crack propagation characteristics (ε_max_, σ_max_ and K_lc_) when the content of hydrated lime and modified foamed asphalt binder with 0.6% SAA is between 15% and 30%, and 5.9% of foamed asphalt binder with 0.6% SAA, respectively.

### 3.7. Optimisation of Foamed Asphalt Binder and Hydrated Lime Content in Terms of Long-Term Durability of FAM

The physical, mechanical and low-temperature properties of FAM AC 8 S depend on the content of hydrated lime in the limestone filler and the foamed asphalt binder with 0.6% SAA. However, as the studies have shown, the quantities of additives used (FA and HL) vary in order to ensure optimal values for the individual parameters tested for FAM AC 8 S. Consequently, in order to optimise the quantities of additives in FAM, desired property functions were utilised [60], which were analysed using Statistica software [73].

The utility of AC 8 S was assessed by assigning a value of 1 to the most desirable response values and a value of 0 to the least desirable response values for the analysed parameters. Intermediate values ranged from 0 to 1, in a linear relationship. The optimisation procedure is described in detail in [73]. The following criteria were applied for the individual FAM AC 8 S parameters:

- Air void content, (V_a_) as per EN 12697-8, (max: 0, min: 1) as per [57];
- Resistance to moisture and frost, (TSR)_,_ as per AASHTO T283 (max: 1, min: 0) as per [59];
- Resistance to permanent deformation (WTS_*AIR*_) as per EN 12697-22 (max: 0, min: 1) as per [60];
- Resistance to permanent deformation (PRD_*AIR*_) as per EN 12697-22 (max: 0, min: 1) as per [62];
- Complex modulus E* as per EN 12697-26 (max: 1, min: 0) as per [79,80];
- Resistance to low temperature cracking (R_−2°C_) as per PANK 4308 (max: 0, min: 1) as per [62];
- Resistance to crack propagation, SCB (ε_max_) as per EN 12697-44 (max: 1, min: 0) as per [81];
- Resistance to crack propagation, SCB (σ_max_) as per EN 12697-44 (max: 1, min: 0) as per [81];
- Resistance to crack propagation, SCB (K_lc_) as per EN 12697-44 (max: 1, min: 0) as per [81].

In order to determine the optimum quantity of foamed asphalt binder with 0.6% SSA and hydrated lime in the limestone aggregate, the significance of the influence of these parameters on the optimisation process was assessed using ANOVA analysis [69], and the results of the calculations are presented using the Statistica software [73] in Table 9.

Based on an analysis of the data presented in Table 9, it can be unequivocally concluded that all the parameters of the FMA AC 8 S analysed have a significant influence on the process of its optimisation, as the *p*-value is less than 0.005.

**Figure 12:**
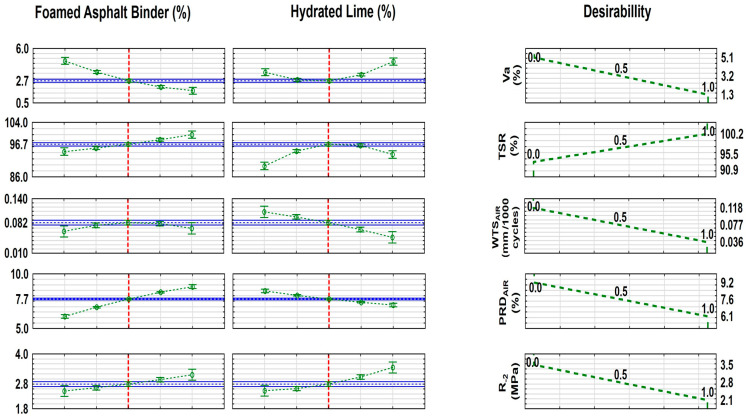

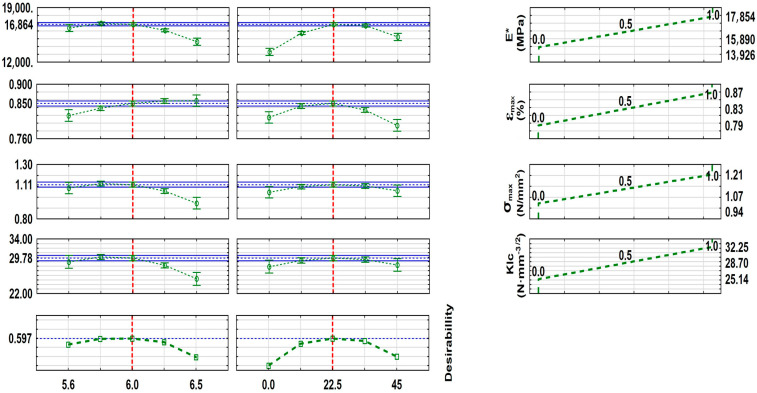
Determinations of the required amount of FA and HL in foamed asphalt mixture AC 8 S for the parameters under analysis.

The results of the optimisation analysis carried out using utility functions are presented graphically using the Statistica software [73] in Figure 12, which also shows a trend of favourable changes in the analysed characteristics of the FAM AC 8 S in terms of FA and HL.

The optimisation results show a varied effect of the quantities of foamed asphalt binder with SAA and hydrated lime on the assessed properties of FAM AC 8 S.

An analysis of the order function of the approximate values shows that the recommended content of foamed asphalt binder with 0.6% SAA content is 6.0%, and the recommended content of hydrated lime replacing the mineral filler is 22.5%. However, taking into account the dosing process and tolerances, the following quantities are recommended: 5.9% of foamed asphalt binder with 0.6% SAA and 30% of hydrated lime. At these percentages of FA and HL, as shown by the optimisation analysis presented in Figure 12, the properties of FAM AC 8 S (FMA_opt_) will continue to meet the recommended values, thereby ensuring the proper functioning of the pavement structural layers and their required durability.

#### 3.7.1. Characteristics of the Physical and Mechanical Properties of FAM AC 8 S with Optimal Amounts of FA and HL

**Figure 13:**
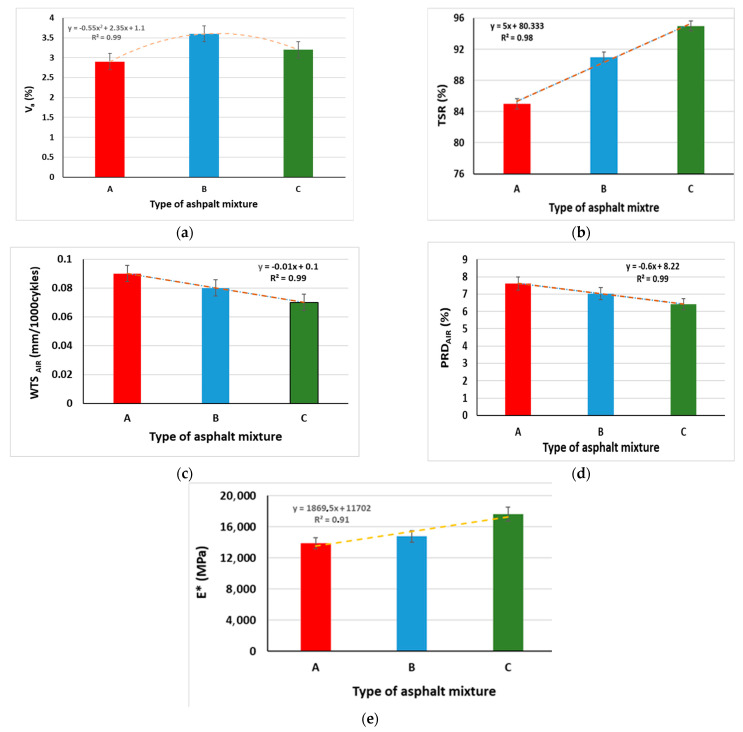
Performance characteristics of the reference AC 8 S with an asphalt binder content of 5.6%, FAM AC 8 S with a foamed asphalt content of 5.6% and FAMopt AC 8 S with an optimal number of additives: (**a**) Va; (**b**) TSR; (**c**) WTS_AIR_; (**d**) PRD_AIR_; (**e**) E*. where A is AC 8 S with 5.6% asphalt binder; B is FAM AC 8 S with 5.6% foamed asphalt binder; C is FAMopt AC 8 S.

A very important element of the analysis aimed at optimising the use of FA and HL additives in relation to the properties of FAM AC 8 S was the comparison of such fundamental material characteristics as void content (V_a_), resistance to moisture and frost (TSR), resistance to permanent deformation (WTS_AIR_ and PRD_AIR_), the combined stiffness modulus E* of the reference AC 8 S with an asphalt binder content of 5.6%, FAM AC 8 S with a foamed asphalt binder content of 5.6% and FAM_opt_ AC 8 S with an optimal amount of foamed asphalt binder with 0.6% SAA and a 30% HL content in the limestone powder. In order to evaluate the asphalt mixtures under analysis, tests were carried out on each parameter using five samples, and the coefficient of variation of the results obtained for each test was less than 9%. The average values of the analysed properties of the asphalt mixtures are shown in Figure 13.

Based on the test results presented in Figure 13, it can be concluded that the properties of FAM_opt_ AC 8 S, which incorporates foamed asphalt binder with 0.6% SAA and hydrated lime, are more favourable than those of the traditional AC 8 S asphalt mixtures produced using HMA technology. In contrast, the properties of FAM AC 8 S containing unmodified foamed asphalt binder lie between those of AC 8 S produced using HMA technology and those of FAM_opt_ AC 8 S. The nature of the changes in the properties of the tested asphalt mixtures in terms of TSR, WTS_AIR_, PRD_AIR_ and E* can be described using linear correlations, which are highly reliable as R^2^ ranges from 0.99 to 0.89. However, the changes in V_a_ with respect to the type of asphalt mixtures were described using a polynomial, as the linear correlation in this case was not very reliable (R^2^ < 0.8).

The use of FAM_opt_ compared with AC 8 S HMA in terms of V_a_, TSR, WTS_AIR_, PRD_AIR_ and E* results in an increase in the values of the analysed characteristics by 10%, 22%, 16%, 12% and 26%, respectively. A key factor in this process is the modification of foamed asphalt binder with a 0.6% SAA additive and the use of hydrated lime in the limestone filler. This beneficial effect of improved values for the analysed properties of FAM_opt_ results from the synergy of the additives used; hydrated lime improves the adhesion of the binder to the aggregate and acts to stiffen the asphalt mixture, whilst the addition of SAA to the foamed asphalt binder affects the elasticity and workability of the asphalt mixture. Thus, the additives used offset their less favourable effects—when used individually—on the analysed properties of the asphalt mixture, whilst simultaneously enhancing them. Of course, the foaming of asphalt binder using water plays a very significant role, as it helps to improve the process of the binder coating the aggregate and enables the production of FMA at 120 °C and its compaction at 100 °C.

#### 3.7.2. Characteristics of the Low-Temperature Properties of FAM AC 8 S with Optimal Amounts of FA and HL

**Figure 14:**
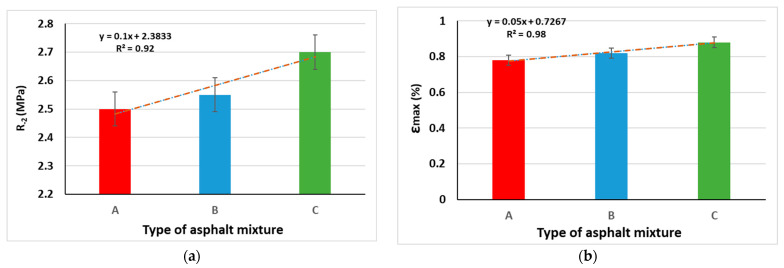

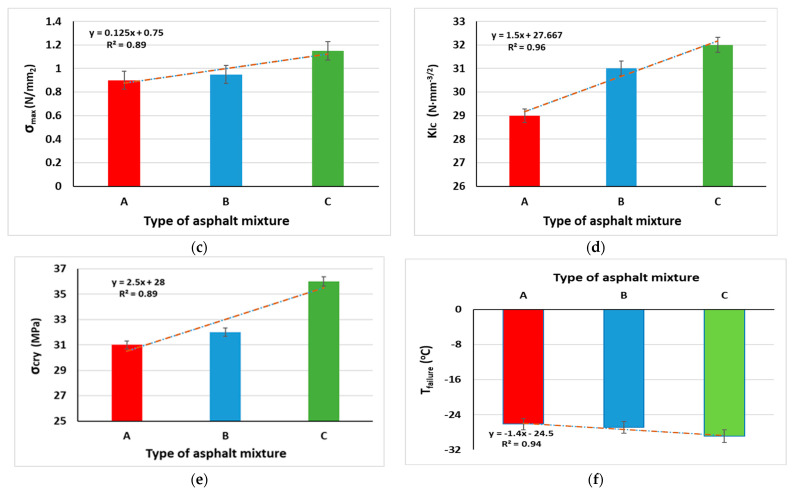
Properties of the reference AC 8 S with an asphalt content of 5.6%, FAM AC 8 S with a foamed asphalt binder content of 5.6% and FAM_opt_ AC 8 S with an optimal amount of additives: (**a**) R_−2_; (**b**) ε_max_; (**c**) σ_max_; (**d**) K_lc_; (**e**) σ_cry_; (**f**) T_failure_. where A is AC 8 S with 5.6% asphalt; B is FAM AC 8 S with 5.6% foamed asphalt; C is FAMopt AC 8 S.

Particular attention was paid during the research process to the low-temperature properties of FAM AC 8 S, which are of significant importance in ensuring its service life over the long term in asphalt pavements in temperate climate zones. Consequently, at this stage of the research, the analysed properties were further expanded to include characteristics determined according to the TSRST methodology [64,82]. To this end, a comparison was made of properties such as resistance to low-temperature cracking (R_−2_), resistance to crack propagation, SCB—strain at maximum force (ε_max_), ultimate stress (σ_max_) and crack resistance (K_lc_), as well as the TSRST characteristics: σ_cry_—maximum thermally induced stress at specimen fracture and T_failure_—the failure temperature of the reference AC 8 S with an asphalt binder content of 5.6%, FAM AC 8 S with a foamed asphalt binder content of 5.6% and FAM_opt_ AC8 S with an optimal amount of foamed asphalt binder with 0.6% SAA and a 30% HL content in the limestone powder. In order to evaluate the asphalt mixtures under analysis, tests were carried out on each parameter using five samples, and the coefficient of variation of the results obtained for each test was less than 7.5%. The average values of the analysed properties of the asphalt mixtures are shown in Figure 14.

An analysis of the results of the low-temperature performance tests shown in Figure 14 indicates that FAM_opt_ AC 8 S, containing foamed asphalt binder with 0.6% SAA and hydrated lime, exhibits more favourable values in comparison with the traditional AC 8 S asphalt mixture produced using HMA technology. In contrast, the properties of FAM AC 8 S, which contains unmodified foamed asphalt binder, lie somewhere in between. The nature of the changes in the properties of the analysed asphalt mixtures in terms of R_−2_, ε_max_, σ_max_, K_lc_, σ_cry_ and T_failure_ can be described using linear correlations, which are highly reliable as R^2^ ranges from 0.98 to 0.89.

The use of foamed asphalt binder with 0.6% SAA and hydrated lime in FAM_opt_ results in an increase in the values of its R_−2_, ε_max_, σ_max_, K_lc_, σ_cry_ and T_failure_ characteristics by 8%, 13%, 22%, 10%, 16% and 11%, respectively, compared to AC 8 S produced using HMA technology. Consequently, FAM_opt_ AC 8 S exhibits greater resistance to low temperatures, thereby ensuring its durability over a longer service life than AC 8 S produced using traditional HMA technology.

## 4. Conclusions

An analysis of the test results for the AC 8 S asphalt mixture, produced using the foamed asphalt mixture technology with foamed asphalt binder with 0.6% SAA and 30% hydrated lime in the mineral aggregate, has led to the following conclusions:

- Foamed asphalt binder with 0.6% SAA and hydrated lime have a significant effect on the void content (V_a_), resistance to moisture and frost (TSR), resistance to permanent deformation characterised by the WTS_AIR_ and PRD_AIR_ indices and the complex modulus of stiffness (E*) of the foamed asphalt mixture, resulting in a favourable change in the analysed characteristics by 10%, 22%, 16%, 12% and 26%, respectively, compared to AC 8 S produced using traditional HMA technology.
- The use of foamed asphalt binder with 0.6% SAA and hydrated lime in FAM_opt_ ensures an increase in the values of its parameters representing non-temperature-dependent characteristics such as resistance to non-temperature-dependent cracking at R_−2°C_, stress at maximum force ε_max_, maximum stress at failure σ_max_, crack resistance K_lc_, stress at cracking σ_cry_ and cracking temperature T_failure_ by 8%, 13%, 22%, 10%, 16% and 11%, respectively, compared to AC 8 S produced using HMA technology.
- The use of hydrated lime and foamed asphalt binder with the addition of SAA improves the material properties of FMA AC 8 S in terms of resistance to climatic factors, ranging from the effects of moisture and frost (TSR) through to resistance to a wide range of stresses at operating temperatures, from high summer (WTS_AIR_, PRD_AIR_) to low winter temperatures (R_−2_, ε_max_, σ_max_, K_lc_, σ_cry_ and T_failure_).
- The beneficial effect of hydrated lime and foamed asphalt binder with the SAA additive on the properties of FAM is the result of the synergy between them; hydrated lime improves the adhesion of the binder to the aggregate and acts to stiffen the asphalt mixture, whilst the addition of SAA to foamed asphalt binder affects the workability of the asphalt mixture. Thus, the additives used, on the one hand, compensate for their less favourable effects as individual additives on the analysed properties of the asphalt mixture and, on the other hand, enhance them.
- The optimisation of the foamed asphalt mixture AC 8 S based on the desirability function enabled the determination of the recommended quantities of hydrated lime in the limestone filler and foamed asphalt binder with 0.6% SAA, at 30% and 5.9%, respectively, ensuring the most favourable levels of the analysed basic properties and low-temperature characteristics.
- Foamed asphalt binder also plays a significant role in ensuring the high-quality material properties of FAM; it improves the process of the binder coating the aggregate and enables the production of FMA at a temperature of 120 °C and its compaction at a temperature of 100 °C.

Based on the results of laboratory tests, it can be concluded that the use of hydrated lime in FAM material with foamed asphalt binder has a significant, positive effect on its properties. Foamed asphalt mixture AC 8 S is resistant to moisture and frost, as well as to permanent deformation and, most importantly, is characterised by high resistance to the adverse effects of low temperatures. These characteristics should ensure the proper performance of the foamed asphalt mixture in the wearing course of an asphalt pavement and its durability over the long service life of the pavement structure.

The results of the laboratory tests obtained justify taking steps to verify them under real-world conditions on a section of road exposed to climatic factors and traffic loads.

## Figures and Tables

**Table 1:** Properties of 50/70 asphalt binder before and after modification (0.6% SAA) [11].

Property	Unit	Testing Method	Asphalt Binder	
			50/70	50/70 + 0.6% SAA
Penetration at 25 °C	0.1 mm	EN 1426 [50]	65.9	70.4
Softening point T_R&B_	°C	EN 1427 [51]	50.4	48.8
Fraass breaking point	°C	EN 12593 [52]	−15.1	−14.2
Temperature plasticity range	°C	-	65.5	63.0
Penetration Index	-	EN 12591 [53]	−0.6	1.4
Expansion ratio (ER)	-	Wirtgen [54]	11	19
Half-life (HLa)	s	Wirtgen [54]	10	21
Optimal foaming water content (OFWC)	%	Wirtgen [54]	2.5	2.5

**Table 2:** Properties of reference asphalt mixture AC 8 S in technologies HMA [56].

Properties	Unit	Testing Method	Result
Air void content in AC, V_a_	%	EN 12697-8 [57]	2.9
Indirect tensile strength, ITS	kPa	EN 12697-23 [58]	1252
Resistance to moisture and frost, ITSR	%	WT-2 2014 [48]	101.9
Resistance to permanent deformation: - WTS_AIR0.15_, - PRD_AIR9.0_	mm/10^3^ cycles (%)	WT-2 2014 [48] (procedure B)	0.09 7.26

**Table 3:** Parameters of the model of the relationships between V_a_ of foamed asphalt mixture AC 8 S and the content of modified foamed asphalt binder and hydrated lime.

Properties	Effect	Regression Coefficient	Std. Error	*p*-Value	−95% Conf. Lmt.	+95% Conf. Lmt.
V_a_ (%); R^2^ = 0.880; MS Res. = 0.101	Intercept	2.704	0.056	<0.001	2.593	2.815
	(1) Foamed asphalt [%] (L)	−2.013	0.074	<0.001	−2.160	−1.867
	Foamed asphalt [%] (Q)	0.512	0.124	<0.001	0.266	0.758
	(2) Hydrated lime [%] (L)	0.669	0.074	<0.001	0.523	0.816
	Hydrated lime [%] (Q)	1.316	0.124	<0.001	1.070	1.562
	1 L ^x^ 2 L	0.781	0.099	<0.001	0.584	0.977
Regression model	V_a_ = 68.158 − 18.425·FA + 1.266·FA^2^ − 0.276·HL + 0.001·HL^2^ + 0.038·FA·HL					

**Table 4:** Parameters of the regression model of the relationship between the TSR of foamed asphalt mixture AC 8 S and the content of the foamed asphalt binder with 0.6% SAA and hydrated lime.

Response	Effect	Parameter	SE	*p*-Value	−95% Cnf. Lmt	+95% Cnf. Lmt
***TSR*** R^2^ = 0.74 MS Res. = 1.596	Intercept	72.342	42.804	0.093	−12.294	156.979
	(1) Foamed asphalt [%] (L)	1.101	14.164	0.938	−26.905	29.107
	Foamed asphalt [%] (Q)	0.386	1.169	0.741	−1.926	2.699
	(2) Hydrated lime [%] (L)	0.542	0.115	<0.000	0.314	0.771
	Hydrated lime [%] (Q)	−0.004	0.000	<0.000	−0.005	−0.003
	1 L * 2 L	−0.048	0.018	0.010	−0.085	−0.011
Regression model	TSR = 72.342 + 1.101·FA + 0.3586·FA^2^ + 0.542·HL − 0.004·HL^2^ − 0.048·FA·HL					

**Table 5:** Parameters of the model of the relationships between WTS_AIR_ i PRD_AIR_ of foamed asphalt mixture AC 8 S and the content of the foamed asphalt binder with 0.6% SAA and hydrated lime.

Properties	Effect	Regression Coefficient	Std. Err.	*p*-Value	−95% Conf. Lmt.	+95% Conf. Lmt.
WTS_AIR_						
R^2^ = 0.660; R^2^ _adj_. = 0.648 MS Res. = 0.101	Intercept	0.080	0.001	<0.001	2.593	2.815
	(1) Foamed asphalt [%] (L)	0.004	0.002	<0.001	−2.160	−1.867
	Foamed asphalt [%] (Q)	−0.011	0.004	0.082	0.266	0.758
	(2) Hydrated lime [%] (L)	−0.039	0.003	<0.001	0.523	0.816
	Hydrated lime [%] (Q)	−0.003	0.004	0.368	1.070	1.562
	1 L * 2 L	−0.003	0.003	0.337	0.584	0.977
Regression model	WTS_AIR_ = 0.08 + 0.004·FA − 0.011·FA^2^ − 0.0339·HL − 0.003 − 0.003·FA·HL					
PRD_AIR_						
R^2^ = 0.952; R^2^ _adj_. = 0.950 MS Res. = 0.029	Intercept	−24.508	5.822	<0.001	−36.022	−12.995
	(1) Foamed asphalt [%] (L)	8.903	1.926	<0.001	5.093	12.712
	Foamed asphalt [%] (Q)	−0.578	0.159	<0.001	−0.893	−0.264
	(2) Hydrated lime [%] (L)	−0.053	0.015	<0.001	−0.084	−0.022
	Hydrated lime [%] (Q)	0.001	0.000	0.266	0.000	0.000
	1 L * 2 L	0.005	0.002	0.046	0.000	0.010
Regression model	PRD_AIR_ = −24.508 + 8.903·FA − 0.578·FA^2^ − 0.053·HL + 0.01·HL^2^ + 0.005·FA·HL					

**Table 6:** Parameters of the model of the relationships between R_−2°C_ of foamed asphalt mixture AC 8 S and the content of foamed asphalt binder with 0.6% SAA and hydrated lime.

Properties	Effect	Regression Coefficient	SE	*p*-Value	−95% Conf. Lmt.	+95% Conf. Lmt.
R_−2°C_ (MPa); R^2^ = 0.735; MS Res. = 0.0311	Intercept	7.2713	5.9701	0.225	−4.53343	19.0760
	(1) Foamed asphalt [%] (L)	−1.8054	1.9755	0.362	−5.71176	2.1007
	Foamed asphalt [%] (Q)	0.1697	0.1631	0.299	−0.15286	0.4923
	(2) Hydrated lime [%] (L)	−0.0471	0.0160	<0.001	−0.07897	−0.0153
	Hydrated lime [%] (Q)	0.0003	0.0001	<0.001	0.00005	0.0003
	1 L * 2 L	0.0088	0.0026	<0.001	0.00363	0.0139
Regression model	R_−2°C_ = 7.2713 − 1.8054·FA + 0.1697·FA^2^ − 0.0471·HL + 0.0003·HL^2^ + 0.0088·FB·HL					

**Table 7:** Parameters of the model of the relationships between E* of foamed asphalt mixture AC 8 S and the content of foamed asphalt binder with 0.6% SAA and hydrated lime (The Complex Modulus of Stiffness is denoted by the symbol E*).

Properties	Effect	Regression Coefficient	SE	*p*-Value	−95% Conf. Lmt.	+95% Conf. Lmt.
E* (MPa); R^2^ = 0.797; MS Res. = 208,768	Intercept	−88,679.4	20,769.37	<0.001	−130,063	−47,295.5
	(1) Foamed asphalt [%] (L)	35,216.1	6872.72	<0.001	21,522	48,910.2
	Foamed asphalt [%] (Q)	−2986.2	567.60	<0.001	−4117	−1855.3
	(2) Hydrated lime [%] (L)	232.0	55.97	<0.001	121	343.6
	Hydrated lime [%] (Q)	−2.3	0.23	<0.001	−3	−1.8
	1 L * 2 L	−16.8	9.08	0.068	−35	1.3
Regression model	E* = − 88,679.4 + 35,216.1·FA − 2986.2·FA^2^ + 0232·HL − 2.3·HL^2^ − 16.8·FB·HL					

**Table 8:** Parameters of the models of the relationship between the crack propagation characteristics (ε_max_, σ_max_ and K_lc_) determined using the SCB procedure of foamed asphalt mixture AC 8 S and the content of foamed asphalt binder with 0.6% SAA and hydrated lime.

Effect	Regression Coefficient	Std. Error	*p*-Value	Confidence Limits	
				−95%	+95%
Variable: ε_max_ (%), 10 mm, R^2^ = 0.607; Pure error RMS = 0.00019					
Intercept	−0.64038	0.471354	0.176	−1.57240	0.29162
(1) Foamed asphalt (%) (L)	0.43093	0.155974	0.006	0.12253	0.73934
Foamed asphalt (%) (Q)	−0.03086	0.012882	0.017	−0.05633	−0.00539
(2) HL (%) (L)	0.00891	0.001270	<0.001	0.00641	0.01143
HL (%) (Q)	−0.00005	0.000005	<0.001	−0.00006	−0.00004
1 L x 2 L	−0.00118	0.000206	<0.001	−0.00159	−0.00077
Regression model	ε_max_ = −0.64038 + 0.43093·FA − 0.03086·FA^2^ + 0.00891·HL − 0.00005·HL^2^ − 0.00118·FB·HL				
Variable: σ_max_ (N/mm^2^), 10 mm, R^2^ = 0.466; Pure error RMS = 0.00319					
Intercept	−7.81177	1.91544	<0.001	−11.5992	−4.02434
(1) Foamed asphalt (%) (L)	3.10367	0.63383	<0.001	1.8504	4.35696
Foamed asphalt (%) (Q)	−0.27006	0.05234	<0.001	−0.3736	−0.16656
(2) HL (%) (L)	−0.00873	0.00516	0.092	−0.0189	0.00148
HL (%) (Q)	−0.00009	0.00002	<0.001	−0.0001	−0.00005
1 L x 2 L	0.00215	0.00084	0.011	0.0005	0.00380
Regression model	σ_max_ = −7.81177 + 3.10367·FA − 0.27006·FA^2^ − 0.00873·HL − 0.00009·HL^2^ − 0.00215·FB·HL				
Variable: K_lc_ (N·mm^−3/2^), 10 mm, R^2^ = 0.465; Pure error RMS = 2.27852					
Intercept	−208.574	51.14248	<0.001	−309.698	−107.450
(1) Foamed asphalt (%) (L)	82.868	16.92338	<0.001	49.405	116.331
Foamed asphalt (%) (Q)	−7.211	1.39766	<0.001	−9.974	−4.447
(2) HL (%) (L)	−0.233	0.13782	0.093	−0.506	0.039
HL (%) (Q)	−0.002	0.00056	<0.001	−0.003	−0.001
1 L x 2 L	0.057	0.02236	0.011	0.013	0.102
Regression model	K_lc_ = − 208,574 + 82.868·FA − 7.211·FA^2^ − 0.233·HL − 0.002·HL^2^ + 0.057·FB·HL				

**Table 9:** Parameters of the models characterising the variables subjected to optimisation (The complex stiffness modulus is marked with the symbol E*).

Dependent Variable	SS Test for the Full Model with Respect to SS for Residual			
	Multiple R	Multiple R^2^	Adjusted R^2^	*p*
V_a_ (%)	0.934	0.873	0.864	<0.001
TSR (%)	0.865	0.748	0.731	<0.001
WTS_AIR_ (mm/10^3^ cycles)	0.778	0.605	0.578	<0.001
PRD_AIR_ (mm)	0.978	0.958	0.955	<0.001
E* (MPa)	0.892	0.797	0.783	<0.001
R_−2_ (MPa)	0.816	0.666	0.644	<0.001
ε_max_ (%)	0.753	0.5672	0.538	<0.001
σ_max_ (N/mm^2^)	0.664	0.443	0.402	<0.001
Klc (N·mm^−3/2^)	0.666	0.442	0.405	<0.001

## Data Availability

The original contributions presented in this study are included in the article. Further inquiries can be directed to the corresponding author.

## Abbreviations

The following abbreviations are used in this manuscript:

FAM	foamed asphalt mixtures
ER	expansion ratio
HLa	half-life
OFWC	optimal foaming water content
SAA	surface-active agent
Va	air void content
WTS_AIR_	rutting plot
PRD_AIR_	proportion rut depth
TSR	tensile strength
R_−2_	resistance to low-temperature cracking
E*	complex modulus of stiffness
4PB-PR	four-point bending beam
SCB	semi-circular bending
ε_max_	strain at maximum force
σ_max_	maximum stress at damage
K_lc_	resistance to cracking
TSRS	thermal stress restrained test
σ_cyr_	failure stress
T_failure_	failure temperature
FA	modified foamed asphalt binder with 0.6% SAA
HL	hydrated lime
